# ANTIPSYCHOTIC MEDICATION USE IN ASSISTED LIVING/RESIDENTIAL CARE: DO ORGANIZATIONAL CHARACTERISTICS MATTER?

**DOI:** 10.1093/geroni/igac059.755

**Published:** 2022-12-20

**Authors:** Sarah Dys, Ozcan Tunalilar, Paula Carder

**Affiliations:** Vital Research, LLC, Los Angeles, California, United States; Portland State University, Portland, Oregon, United States; Portland State University, Portland, Oregon, United States

## Abstract

This study investigates how assisted living/residential care (AL/RC) and memory care (MC) contexts are associated with the prevalence of antipsychotic medication use (APU). Primary data were collected from a statewide representative sample of AL/RC settings through the Oregon Community-Based Care study from 2017-2019 and combined with publicly available administrative data. Framed by Donabedian’s model of care quality, we examine associations among 90-day prevalence of APU, organizational, care process, and AL/RC resident population characteristics using random intercepts regression models. Every licensed AL/RC setting in Oregon receives an annual mailed survey to provide aggregate resident demographics, health acuity, health service use, payment type and organizational policies. Organizational measures (e.g., profit status, license type, geographic designation) were collected from state websites. The average 90-day prevalence of APU among all Oregon AL/RC settings is 30.7%, though rates differ by MC endorsement (23.9% in AL/RC and 42.7% in MC). Compared to care processes and resident population characteristics, organizational characteristics were associated with larger magnitudes of difference in rates of APU. Nonprofit settings were associated with lower rates of APU in both AL/RC (β= -4.4 percentage points, [95% CI: -8.4, -0.4]) and MC (β= -12.4, [95% CI: -21.2, -3.6]. Compared to low-Medicaid settings, settings with very high proportions of Medicaid residents were associated with higher prevalence of APU, +8.9 in AL/RC (95% CI: 1.7, 16.1) and +11.0 in MC (95% CI: 2.3, 19.8). Policymakers might consider how organizational resource capacity influences APU in AL/RC settings, especially if APU prevalence is treated as a quality indicator.

